# Process evaluation of the Yéego! Program to increase healthy eating and gardening among American Indian elementary school children

**DOI:** 10.1186/s12889-024-17689-6

**Published:** 2024-01-19

**Authors:** Heather Wilcox, Sonia Bishop, Brandon Francis, Kevin Lombard, Shirley A. A. Beresford, India J. Ornelas

**Affiliations:** 1 University of Washington, Box 351621, Seattle, WA 98195 USA; 2 Fred Hutchinson Cancer Research Center, Seattle, USA; 3 New Mexico State University, Las Cruces, USA

**Keywords:** Process evaluation, Fidelity, Implementation, School-based intervention

## Abstract

**Background:**

American Indian children are at increased risk for obesity and diabetes. School-based health promotion interventions are one approach to promoting healthy behaviors to reduce this risk, yet few studies have described their implementation and fidelity. We conducted a qualitative process evaluation of the Yéego! Healthy Eating and Gardening Program, a school-based intervention to promote healthy eating among Navajo elementary school children. The intervention included a yearlong integrated curriculum, as well as the construction and maintenance of a school-based garden.

**Methods:**

Our process evaluation included fidelity checklists completed by program staff and qualitative interviews with program staff and classroom teachers after the intervention was implemented. We used content analysis to identify themes.

**Results:**

We identified several themes related to evidence of delivery adherence, program satisfaction, and lessons learned about delivery. Intervention staff followed similar procedures to prepare for and deliver lessons, but timing, teaching styles, and school-level factors also impacted overall implementation fidelity. Teachers and students had positive perceptions of the program, especially lessons that were highly visual, experiential, and connected to Navajo culture and the surrounding community. Teachers and program staff identified ways to enhance the usability of the curriculum by narrowing the scope, relating content to student experiences, and aligning content with school curriculum standards.

**Conclusions:**

The program was implemented with moderately high fidelity across contexts. We identified areas where modifications could improve engagement, acceptability, efficacy, and sustainability of the program. Our results have implications for the evaluation and dissemination of school-based health interventions to promote healthy eating among children, especially in American Indian communities.

## Background

American Indian populations in the United States are at increased risk for several chronic conditions related to healthy eating, such as obesity, type 2 diabetes, cardiovascular disease and cancer [1–5]. Healthy eating patterns are often established in childhood, and these behaviors can contribute to decreased risk for disease in adulthood. Many American Indian communities, including the Navajo Nation, are working to reduce rates of childhood obesity, but face significant barriers such as household food insecurity and limited access to healthy foods [1, 6–9]. Fresh fruits and vegetables are particularly difficult for families to access on the Navajo Nation due to their high cost and the need to travel long distances to purchase them [10, 11]. School-based interventions have been identified as an effective approach to promoting healthy behaviors in childhood [12–15]. Studies have shown that school-based interventions which incorporate nutrition education and gardening can increase healthy eating behaviors among elementary school children [14, 16–19]. Interventions to improve healthy eating in American Indian communities have also noted the importance of including traditional foods and food sovereignty [20–22].

The Yéego! Healthy Eating and Gardening Program (Yéego! Program) is a school-based intervention to promote healthy eating behavior among elementary school children on the Navajo Nation [23, 24]. The Yéego! Program combines a school garden with a culturally relevant nutrition and gardening curriculum designed for third- and fourth-grade students. The intervention was developed by a research team from New Mexico State University, Diné College and the Fred Hutchinson Cancer Research Center who collaborated with community leaders in the Navajo Nation to design the curriculum content. During the 2016–2017 school year, the Yéego! Program was pilot tested at Dream Diné Charter School on the Navajo Nation. Lessons learned from that pilot were then incorporated into a revised curriculum, which was evaluated in a randomized controlled trial in six elementary schools in the Tsaile and Shiprock regions of the Navajo Nation.

Previous evaluation studies have identified contextual challenges to the implementation of school-based health interventions, including issues related to fidelity [14, 25–28]. Fidelity can be particularly important in evaluating school-based interventions, due to the variation among teachers, classrooms, schools, and education systems. For example, role ambiguity in the intervention design, limited time, knowledge and resources among school staff, and the impact of teacher absences [29–31]. Only a few studies have reported findings of process evaluations of school-based gardening and eating interventions with mixed results [22, 32]. One study noted that low implementation fidelity was due to the extensive preparation time needed [32]. Even when schools received the necessary funding and materials, the preparation involved made it difficult to implement them without additional assistance [32]. Another study in the Netherlands found that students’ preferences influenced teachers’ implementation of intervention elements [33]. Furthermore, a recent review of nutrition intervention strategies among American Indian youth has called for more research to better understand the effectiveness of school-based interventions [34]. Therefore, there is need for further research to better understand the contextual factors that shape intervention implementation fidelity so that we can design and disseminate effective school-based health interventions.

We conducted a process evaluation of the Yéego! Program in order to describe implementation across different contexts and identify aspects of the delivery that could be improved in future iterations of the program. Results were intended to help interpret outcomes of the intervention trial, suggest ways to improve the program and inform the implementation of similar programs.

## Methods

### Description of intervention delivery

The intervention trial took place in two communities (Tsaile and Shiprock). One school in each community received the Yéego! Program during the first year of the study (2019–2020), while two schools in each community served as controls. Based on the study design, control schools were supposed the receive the intervention the following year [24]. However, due to the COVID-19 pandemic we were unable to offer the program in person, and instead provided the schools with the curriculum and resources to build a school-based garden for later use. The curriculum was comprised of 17 lessons: eight gardening lessons, eight healthy eating lessons, and one combined lesson to conclude the program (Table 1). The program was delivered during classroom time alongside teachers’ other curricula. The process evaluation assessed implementation fidelity at the two schools that received the intervention in the first year (see site characteristics in Table 2). Five intervention staff (three at Site A and two at Site B) with expertise in agriculture, gardening education, and/or nutrition delivered the lessons. Gardening lessons at Site A were delivered by one member of the research team who was Navajo. Healthy Eating lessons at Site A were delivered by two non-Navajo staff from the local Indian Health Services clinic. Gardening lessons and Healthy Eating lessons at Site B were each delivered by agricultural extension staff, both of whom were Navajo. While intervention staff had primary responsibility for delivering the lessons, the study intended to gradually transition delivery to classroom teachers to support long term sustainability. Lessons occurred every two weeks, alternating Healthy Eating lessons with Gardening lessons. Because the COVID-19 pandemic caused schools to close abruptly in March 2020, neither school was able to complete all of the lessons in the intervention. A summary of intervention delivery at each site is presented in Table 2.

**Table 1:** Curriculum Topics in the Yéego! Healthy Eating and Gardening Program

Lesson #	Healthy Eating	Gardening
1	Introduction and Kitchen Safety	Introduction to the Garden
2	Reading a Recipe	Maintaining the Garden
3	Whole Foods and Nutrition	Food Preservation and Seed Saving
4	Eating for Energy	Soil and Compost
5	Fruits and Vegetables	Water in the Garden
6	Traditional Foods and Food Sovereignty	Plant Parts and Life Cycle
7	Healthy Meals and Healthy Families	Native Plants and Navajo Ecology
8	Garden to Table	Getting Ready to Plant in the Garden
9	Garden Celebration (combined lesson)	

**Table 2:** Site Characteristics and Overview of Intervention Delivery

Characteristics	Site A	Site B
Number of 3rd grade classrooms^a^	3	3
Number of 3rd grade teachers interviewed	3	1
Median students per 3rd grade classroom^a^	15	16
Number of 4th grade classrooms^a^	3	2
Number of 4th grade teachers interviewed	3	1
Median students per 4th grade classroom^a^	15	25
% of students who are American Indian or Alaska Native^a^	81%	96%
% of students who understand Navajo language^a^	40%	59%
**Gardening Lessons**		
Number of intervention staff + *(support personnel)*	1 *(0–1)*	1 *(0)*
Number of lessons delivered	8	6
**Healthy Eating lessons**		
Number of intervention staff + *(support personnel)*	2 *(0–1)*	1 *(0–1)*
Number of lessons delivered	8	8
**Total lessons**		
Total number of lessons delivered	16	14
% of intervention completed	94%	82%

^a^ Includes all classrooms which participated in the randomized controlled trial

### Data collection

We conducted semi-structured individual interviews with teachers and intervention staff, and reviewed fidelity checklists that were completed by intervention staff following the delivery of each lesson. Interviews were conducted with intervention staff (*N* = 5) and classroom teachers (*N* = 11) whose classrooms received the intervention. Contact information could not be obtained for two of the teachers who were no longer employed at Site B, and they were excluded from the present study. All intervention staff gave consent to participate in an interview (*n* = 5), and two of the intervention staff who co-facilitated Healthy Eating lessons at Site A participated in a single interview together. Of the nine teachers contacted, eight agreed to participate (*n* = 8). All interviews were conducted by a trained interviewer in Fall 2020 via Zoom. The study was approved by the human subjects review boards of the Fred Hutchinson Cancer Research Center, New Mexico State University and the Navajo Nation.

#### Fidelity checklists

Intervention staff completed a checklist after each lesson that included attendance; lesson duration; whether they had the necessary supplies; and which lesson components they covered. Because intervention staff delivered the lesson to two or three classrooms in a visit, they would most often complete a single checklist to summarize lesson delivery for all of the classrooms. Research staff reviewed checklists weekly to clarify any questions and maintain data quality.

#### Interviews with intervention staff and classroom teachers

The checklist was used to inform the creation of two interview guides, one for intervention staff and one for classroom teachers. Interview guides consisted of open-ended questions related to factors that shaped program delivery at each school, and identify lessons learned from implementation. Intervention staff were also asked about how they prepared for lessons; challenges to delivering lessons; suggestions for improving the curriculum; interactions with students, teachers, and administrators; and how to support teachers in delivering the program. Classroom teachers were asked about their expectations for the curriculum; level of involvement in delivery; perceptions of student engagement; suggestions for improving the curriculum; and conditions that would enable them to implement the program themselves. Participants were emailed a curriculum overview to reference during the interview. Interviews lasted approximately 60 min. Participants received a $30 gift card in appreciation of their time.

### Analysis

All interviews were recorded and the interviewer wrote detailed notes for each interview. The notes were compiled into a document organized by interview question. The interviewer (HW) and another study team (IO) member used a deductive thematic analysis approach [35]. They reviewed interview notes and fidelity checklists together to identify consistent themes within the data. Themes were defined as concepts that occurred more than once, and which specifically related to similarities and differences in program delivery across sites, classrooms, and intervention staff; perceptions of the program; and conditions that would support teachers to deliver the intervention. The same two members of the research team organized preliminary themes into broad categories in Microsoft Word documents and refined them over multiple iterations of discussion, interpretation and review. They also selected salient participant quotations to illustrate the themes. Themes and quotations were shared with the research team and community partners throughout this iterative process to assist with interpretation.

## Results

We organized themes into three categories: (1) evidence of adherence in delivery, (2) elements supporting program satisfaction, and (3) lessons learned about delivery. Table 3 provides an overview of themes identified in each of the three categories. The sections that follow provide a description of each theme and example quotations from teachers and intervention staff.

**Table 3:** Themes Identified in Data from Teachers and Intervention Staff, Organized by Category

Evidence of Adherence in Delivery	Elements Supporting Program Satisfaction	Lessons Learned About Delivery
• All intervention staff had a preparation routine • Intervention staff used the lesson plan, but variations were common • Intervention staff and teachers did not co-teach lessons as planned • Level of engagement with the school garden was variable across classrooms	• Visual, experiential, and cultural aspects of the lessons maximized student engagement and understanding • Students were engaged by lessons that related to their families and communities	• The scope of curriculum content was ambitious for the timeframe • Lessons could be more inclusive of diverse student needs and life experiences • The curriculum should align with standards and existing curricula • Several barriers impeded the transition of lesson delivery from intervention staff to teachers

### Evidence of adherence in delivery

#### All intervention staff had a preparation routine

Preparation included reviewing the lesson plan, communicating with the teachers about the plan by phone or email, and purchasing and preparing materials needed for the lesson. Overall, intervention staff estimated spending between 2 and 8 h preparing for each lesson.

#### Intervention staff used the lesson plan, but variations were common

Staff relied on the lesson plan to guide content delivery and time management, trying to follow the structure as closely as possible for every classroom. However, all noted changing or omitting some elements due to time constraints. Intervention staff also had different teaching styles and commonly adapted lesson delivery to increase student engagement, facilitate comprehension, and stay within the time limitations. As one staff described:

We had to adapt the curriculum a little bit to the kids…. I would look at the curriculum and go okay, I want this one to be, like, more of a Q&A interactive discussion, so the kids are more engaged. So, how do I need to take the points and transform them into questions so that the kids can understand it more?

Similarly, another participant indicated they began each lesson as a traditional teaching, and they often spoke with students in Navajo language to hold their attention.

Key elements of every lesson were included across sites, but not always implemented with consistency. For example, student journals were intended to be used for review and reflection at the conclusion of every lesson. Journals were more regularly integrated into the lessons at Site A than at Site B, but even with regular use, one teacher at Site A noted that there was inconsistency in how students were instructed to use them, which limited their effectiveness.

#### Intervention staff and teachers did not co-teach lessons as planned

The implementation process was intended to be a gradual transition of lesson responsibilities from intervention staff to teachers, but the transition did not occur in any of the classrooms at either site. All of the teachers assisted intervention staff by managing the classroom and monitoring students throughout the lessons, but only about half of the teachers at each site got more involved. Teachers supported intervention staff by assisting with the activities, reading stories, distributing materials, and providing individualized support to students. They also made efforts to incorporate material from the program lessons into their other curricula, but generally did not co-teach program lessons. Intervention staff tried to encourage participation by talking with the teachers before class about what they would cover, and asking classroom teachers to help with certain portions. This helped engage teachers in some of the activities, but most expected intervention staff to lead the teaching.

#### Level of engagement with the school garden was variable across classrooms

Teachers at both sites expected that students would be able to engage with the school garden more than they did. Students enjoyed spending time in the garden, but that opportunities for hands-on learning were limited, especially for the larger classrooms. All of the classrooms at each site shared one communal garden plot, which made it difficult to ensure all students could equally participate in the gardening activities. Teachers and intervention staff suggested that student engagement could be improved if each classroom had responsibility for a small section of the garden space, rather than all classrooms sharing one larger plot.

### Elements supporting program satisfaction

Teachers and intervention staff at both sites described high levels of engagement and satisfaction among students. Teachers reported that students looked forward to “Yéego” days and saw value in the curriculum. The following themes highlight strengths of the curriculum.

#### Visual, experiential, and cultural aspects of the lessons maximized student engagement and understanding

Intervention staff and teachers consistently described students as being most engaged with lesson content that was highly visual, hands-on, and had tangible connections to students’ life experiences, people in their community, and Navajo culture (Table 4). Teachers noted the emphasis on Navajo cultural aspects as being one of the program’s biggest strengths. Cultural aspects resonated with students, and teachers appreciated the ways in which culture was tied into lessons in appropriate and engaging ways. As one teacher described:

**Table 4:** Example Quotations for Themes Identified as “Elements Supporting Program Satisfaction”

**Visual, experiential, and cultural aspects of the lessons maximized student engagement and understanding**
“Everything needs to be experiential, or hands-on, have a visual support with it. Lecturing to 3rd graders who are learning English is just going to be in one ear and out the other.” (Teacher, Site A)
“The kids LOVED [the guest speaker]. Obviously, her background in teaching and Diné language was a huge plus, but the kids were excited to count and share their Diné vocabulary with her. We made blue corn pancakes together, and the kids really enjoyed watching the whole process. Many of the kids wanted copies of the recipes to take home to their parents.” (Intervention Staff, Site A)
**Students were engaged by lessons that related to their families and communities**
“There was a recipe given, which was shared with all the students, and I think out of my 17 students, three of the students actually used the recipe at home and shared that their family made it.” (Teacher, Site A)
“I think one of the coolest things was when the kids, like, started being able to ask the questions that were actually important to them and like, ‘Oh my gosh, like I bought that at the flea market’ or whatever or, ‘My grandma makes those pancakes,’ just kind of like, making connections that feel real to them. So I thought that was really good.” (Teacher, Site A)
“They had good discussion.. about growing their own food, too. Because I remember they talked about.. how they, or their neighbors, or their family grow corn and some of the traditional foods.” (Intervention Staff, Site A)

“The ability of certain lessons to have the kids feel…their culture, history, and families were something worth studying—something worth exploring and understanding.. it was an absolute joy to witness for me and really built on the kids’ strengths.” (Teacher, Site A).

Another teacher noted:

“I have a lot of kids who are really interested in planting and…anything in their traditional backgrounds, so I would say anything that they can either build or make, or can be tied in with, like, a traditional story really went over super well. Because like, the more connections they can make to their own lives would just be, like, a lot stronger for them.” (Teacher, Site A).

Students especially enjoyed activities that engaged their senses. Preparing and eating food were consistently discussed as highly engaging lesson aspects, as was time spent in the school garden, where students “could get dirty and plant things.” A teacher described how students reacted after tasting the pickled vegetables they made, which smelled strongly of vinegar:

“[The students] finally took a bite into it…and they were just amazed! Like, just hearing that and just seeing their faces, just, surprised—it was kind of like a magical moment for them…. Those activities were a lot of fun for the students.” (Teacher, Site B).

Teachers suggested that incorporating more foods from the garden and integrating them into the snacks provided during Healthy Eating lessons.

Teachers also stressed the importance of experiences that help translate abstract concepts into concrete understandings. One teacher noted that students responded well to lessons built around an anchoring experiential activity. For example, in a lesson about the water cycle and how plants transport water, students put celery stalks in food-colored water and periodically observed the color progressing upward toward the leaves. As one of the intervention staff described: “They wanted to take the celery experiment home…. I think that resonated with them—was actually seeing the phloem and everything, the process. It was great for them.”

#### Students were engaged by lessons that related to their families and communities

Intervention staff noted that students were more engaged when they could connect the lesson content to their community (Table 4). Some teachers felt that the program could more effectively bridge the gap between students and their families, since parents or other family members are usually making food decisions in the house. An important facilitator in bridging students’ learning to their family was using activities that students could bring or recreate at home. One of the intervention staff stated that students often asked questions about how they could do certain activities at home, and “they always want to show their parents what they’re doing.”

### Lessons learned about delivery

#### The scope of curriculum content was ambitious for the timeframe

Nearly all of the teachers and intervention staff indicated that time was a challenge to completing the curriculum as planned. Most intervention staff expressed that it was difficult to cover all of the expected content in the allotted time for each lesson, and they often felt rushed. Common issues were needing time to set up, arriving late, students needing time to transition into the lesson, or students’ prior activities running late. There were occasions at both schools when activities had to be shortened, and one of the intervention staff noted that activities generally took longer for third-grade classes than for fourth-grade classes.

One teacher suggested narrowing the scope of the curriculum, both in terms of the amount of content in individual lessons and in terms of the breadth of learning goals for students (Table 5). They expressed that content delivery was “too much, too fast” and that students would benefit from more time for deductive reasoning and asking questions to deepen their understanding. Some teachers suggested that keeping the lessons to 45 min would make it easier for students to stay focused, and leave time for setup, cleanup, and students to transition from their previous class or activity.

**Table 5:** Example Quotations for Themes Identified as “Lessons Learned About Delivery”

**The scope of curriculum content was ambitious for the timeframe**
“And there were times, yeah, that we had to kind of rush through things and finish up because it was time for them to switch to another classroom.. So that was probably our biggest thing, is that getting everything done within the 45 minutes to an hour timeframe before my class had to move on to go to do something else.” (Teacher, Site A)
“We need more time for these activities because sometimes we had to cut out the last activity, even though it was 10 minutes; 10 minutes in third-grade time is way different than fourth-grade time.” (Intervention Staff, Site A)
**Lessons could be more inclusive of diverse student needs and life experiences**
“One major blind spot that I perceived—and I know that other teachers did, too—was the reality that a lot of kids have food insecurity.. For kids who are poor, calories are calories.” (Teacher, Site A)
“I have kids with behaviors. I have kids who need me to do, you know, scaffolding with them, like visual supports and things like that. So a lot of things take a really long time in elementary school, and I, I don’t necessarily get the impression that, like, the curriculum was set up with that in mind.” (Teacher, Site A)
“Add other things that could help the kids in that area, you know, with their needs.. Some of them are cooking, they’re cooking for the parents, they’re helping out, they’re taking care of their parents, that kind of thing.” (Teacher, Site A)
**The curriculum should align with standards and existing curricula**
“It didn’t go against what we were doing. I would say to truly support [standards], we would have to incorporate math, we would have to incorporate more reading type of things, but it was mostly just science.. I would say the Gardening lessons did a really good job to our cultural standards, though.. I thought that was great because that’s a challenge for us.” (Teacher, Site A)
“We adjusted our standards to correlate with the Yéego gardening standard. So, whatever standard they were teaching that day…we correlated that with our standard, and we made it fit into ELA, math, social studies, science.” (Teacher, Site A)
“Kind of like making dough, you’re putting everything together and you’re making bread and tortilla, and it came out just right. Because when we did that, it kept reinforcing the healthy eating and then it tied in with our standards, and it worked out well…” (Teacher, Site B)
**Several barriers impeded the transition of lesson delivery from intervention staff to teachers**
“So they did tell us, like, they told us from the get-go, ‘We’re providing this lesson because the end goal is that we do this for the year, and that you pick it up and continue it after seeing the modeling.’.. I knew that I wasn’t to check out; this wasn’t, you know, a guest speaker time. This was a time that I’m bringing in a guest speaker from the community to present something, but we work together.” (Teacher, Site A)
“They told us it was going to be.. a gradual release for us, like they would be taking more of the burden in the beginning for teaching, and then at the end we would be. Honestly, just with all the other stuff that I had to do, it was an unrealistic expectation, I think, of all the teachers to have all the necessary, like, preparation and just, like, an understanding of the content—like I do not know how to garden.. I don’t know that much about nutrition in the way that they were teaching it.” (Teacher, Site A)
“Because I really didn’t know what was going on, I was merely just going to facilitate—or monitor students, and then kind of just learn along with them, because I didn’t really know what was going to happen.” (Teacher, Site B)
“I only know the theoretics of actually gardening, but to actually implement it.. in general I don’t really know too much about gardening itself.. And when I was speaking with [another teacher] last year I asked her too, I was like, ‘Are you a big gardener?’ She gardened a little but not too—I don’t think she gardened *that much* to really be able to teach it, I think is what she said? So both of us were still really not at ease with teaching gardening, because we didn’t feel like we were up to, up to par with it.” (Teacher, Site B)
“I probably wouldn’t have done it without [the intervention staff]. I don’t think this is doable, like, alone. Only because the money makes things possible, and thoughts, ideas, coming from people are very helpful.” (Teacher, Site B)
“Teachers that have no clue about ag[riculture] and farming, it would be a little bit hard for them to kind of, actually teach this, I think.” (Intervention Staff, Site B)

#### Lessons could be more inclusive of diverse student needs and life experiences

Teachers and intervention staff both pointed to ways in which some lessons were not adequately responsive to students’ lived experiences. Certain aspects of Healthy Eating lessons, in particular, needed to be more responsive to different family and household structures as well as the realities of food insecurity and being low-income. One teacher suggested greater emphasis on traditional and less expensive foods. They also noted that many students do not have control over the timing and content of their meals at home. The intervention staff suggested that “using examples… from school lunch or school breakfast,” to be more inclusive.

Third- and fourth-grade teachers at both sites generally felt that lesson content was suitable for the grade level, but students in Site A classrooms had diverse learning needs that were not always met. Many students were English-Language Learners and/or low-literacy learners. To support their learning needs, the curriculum could provide more descriptive visual content, especially colorful photos and videos to support students’ comprehension of abstract concepts. Third-grade students were also in various stages of fine motor skills development, which impacted their ability to engage with lesson elements that involved writing, using scissors, or cutting food with a knife. To improve inclusiveness, teachers recommended sentence stems for writing activities; more explicit instruction on how to hold a knife; using a softer object like a banana to practice knife skills; and using pre-cut worksheets rather than having students cut out the pieces. Other learning supports that students needed which the curriculum did not provide were: word banks to help with spelling; time dedicated to introducing vocabulary; explicit written and verbal instructions at the beginning of every activity; and flexibility within the lesson plan to provide extra time for students to complete activities.

Finally, a few teachers across both sites spoke to a need for the curriculum to be built on “a more realistic understanding of what the kids already know and what they don’t know” in order for it to be responsive to diverse community contexts. One teacher noted that students may not have had previous exposure to gardening or farming. Another teacher suggested that it may be more relevant to talk about gardening in containers or raised beds at home, since families may not have the resources to prepare the ground for planting in their area.

#### The curriculum should align with standards and existing curricula

Teachers had varying perceptions of how well the program supported the learning outcomes and standards at their respective schools. Overall, teachers noted that the curriculum best supported standards for science, Navajo culture, health education and language arts. However, it lacked sufficient depth and individualized support in order to meet standards for reading, writing, and math. Teachers wanted a more explicit structure around student work expectations, in order to align the content with learning standards in their lesson plans. Multiple teachers recommended developing a “student edition” of the curriculum, or workbook that contains: handouts; explicit step-by-step written instructions for every activity; descriptive visuals and colorful pictures; vocabulary words and word banks; and a table of contents. A student workbook would give teachers foresight about how students will engage with the curriculum and how learning standards will be met, and it would also give students more ownership of their learning. More explicit structure would also help teachers integrate the curriculum with the other topics they teach, facilitating stronger support of learning standards in general.

#### Several barriers impeded the transition of lesson delivery from intervention staff to teachers

These included variable role expectations, insufficient content expertise among teachers, and onset of the COVID-19 pandemic. About half the teachers expressed an understanding that the intervention was planned to gradually transition from the intervention staff to the teachers, but others either did not perceive lesson delivery as part of their role or did not have enough clarity on their intended role (Table 5). Teachers who expressed an understanding of the transition also tended to be more engaged in helping with the lessons. Some teachers expressed that they did not have the expertise needed to teach the curriculum, nor the time to sufficiently prepare and understand the content (Table 5). Neither of the sites were able to finish the curriculum due to schools closing at the onset of the COVID-19 pandemic, and two of the teachers indicated this as a primary reason for not fully transitioning lesson delivery responsibilities.

When discussing how they might implement the curriculum independently in the future, teachers noted that time and money were significant barriers. The curriculum requires a large quantity of materials, which would be costly. They also expressed concerns about the amount of preparation time required to purchase and prepare fresh fruits and vegetables, secure activity materials, and become familiar with the lesson content. Intervention staff expressed similar concerns about the preparation time and money required, and concern about teachers’ limited gardening knowledge (Table 5). Without sufficient funds and time to prepare, lessons would not be carried out as intended. Teachers suggested creating a student workbook to clarify what the student work expectations are, and videos that model how to teach each the lessons.

## Discussion

Our process evaluation indicated that the program was delivered consistently across contexts. This was because most intervention lessons were delivered at both schools, with only slight variations to intervention content and delivery. While teachers and students were highly receptive to the program, some teachers were hesitant about delivering it independently, and identified areas where student engagement could be improved. Our findings pointed to curriculum strengths and lessons learned that can inform future implementation of this program and similar school-based interventions.

While the program was implemented as planned at both sites, the intensity of experiential components varied across contexts. Previous school-based studies have found that active participation in hands-on gardening activities throughout the entire plant growth process is important to the efficacy of youth gardening programs [36, 37]. Experiential learning through planting, maintaining, harvesting, and preparing traditional foods promotes a sense of ownership and pride among students that supports the success of gardening programs [37, 38]. Therefore, increasing the intensity of gardening activities might provide students with more opportunities to practice the skills, knowledge, and behaviors taught in the curriculum. These changes are also aligned with the intervention’s theoretical underpinnings in Social Cognitive Theory. Ultimately this may also enhance the program’s effectiveness [39–41].

The long-term goal of the program is for teachers to be able to implement the curriculum independently; however, similar to other school based interventions, we identified several barriers to long-term sustainability [35, 42–47]. Studies indicate that more intensive training can support teachers’ confidence and ability to deliver lessons with fidelity [48, 49]. Other school garden programs use community volunteers—including parents and grandparents—as well as specialists from local organizations to assist with garden maintenance and lesson delivery [46, 50]. Our results indicated that incorporating more teaching supports would enhance the acceptability of the program. For example, teachers or other school staff could be provided training on the curriculum during summer break as a part of their continuing education and professional development. Teachers aides or other staff could also be used to assist with both the lessons and gardening maintenance. While teachers had mixed opinions on receiving formal training on the curriculum, most agreed that having someone help to deliver the lessons was necessary. These additional supports would require additional funding to schools to support staff time.

Our findings also suggested a need for stronger alignment with learning standards and teachers’ existing curricula. Several studies indicate that strong curriculum integration aids both the feasibility and sustainability of high-fidelity implementation [16, 48, 51–53]. School-based studies have found that teachers are more likely to prioritize implementation if they can incorporate the program activities into their teaching plans (Day et al., 2019). Integrating gardening activities throughout the broader curriculum can maximize learning opportunities, support maintenance of school-based gardens and ultimately have a positive impact on fruit and vegetable consumption [15, 32, 46]. Involving teachers in the development of program activities may also strengthen linkages between the program and existing curricula.

As the first study to evaluate the implementation process of a school-based gardening program on the Navajo Nation, our study adds to the literature on the effective delivery of school-based interventions to promote healthy eating behaviors among Navajo youth. Based on our findings, we identified several ways to modify program content and delivery to enhance its acceptability and potential efficacy (outlined in Table 6).

**Table 6:** Recommended modifications to the Yéego! Program content and delivery

Recommendation	Suggested Actions
Narrow the scope of the curriculum	• Focus on a small number of overall learning objectives. • Make every lesson deliverable in 45 min or less, plus setup and cleanup time.
Make the curriculum more immediately usable	• Provide a well-organized online interface and videos that model how to teach each of the lessons. • Create a student workbook with handouts in the order students will use them; explicit step-by-step written instructions for every activity; descriptive visuals and colorful pictures; vocabulary words and word banks; and a table of contents.
Reduce or offset costs associated with program materials	• Make more of the activities team-based to reduce the quantity of materials needed. • Foster relationships with community members, businesses, and organizations, who may donate time and resources to the program.
Support capacity of teachers to deliver lessons	• Involve community members, parents, and grandparents to help with lesson delivery and classroom management. • Invite them to contribute their skills, knowledge, and experiences to the lessons.
Achieve stronger alignment with learning standards	• Collaborate with teachers to further align curriculum with science and Navajo culture learning standards. • Provide teachers with example lesson plans that illustrate how gardening and healthy eating activities can be incorporated into their existing curricula.
Maximize content relevance and level of engagement from teachers and students	• Continue to emphasize visual, experiential, and Navajo cultural aspects of lessons, and reconsider how didactic content can be delivered (e.g., use videos to explain foundational concepts). • Assign classrooms their own section of the garden to increase students’ active participation. • Increase the intensity of hands-on gardening and healthy eating activities. • Include time in the lessons for teachers, students, and other community members to contribute their own knowledge and connect the content to their own lives and communities.

### Lessons learned

The study used self-report methods for data collection, which was a time- and cost-effective alternative to observational methods. A limitation of using self-report methods in fidelity research is the potential for desirability bias, which could result in over-estimation of fidelity. Additionally, the level of detail reported in the fidelity checklists was inconsistent across staff, which may have impacted conclusions drawn across sites. Finally, we had some missing data as we were unable to reach some of the teachers and some participants may have found it difficult to recall implementation details due to the time between the intervention and the interviews.

## Conclusions

This process evaluation aimed to describe the fidelity of a school-based health promotion intervention across different contexts and identify elements that could be improved. Based on our assessment of program delivery at two schools on the Navajo Nation, we concluded that the program was implemented with moderately high fidelity across contexts. As noted by teachers and program staff, several factors were identified that could inform future implementation and research to improve healthy eating among elementary school children across the Navajo Nation.

## Data Availability

The datasets generated and/or analysed during the current study are not publicly available to ensure the protection of members of the Navajo Nation and their data sovereignty but may be available from the corresponding author on request.
